# GBP2 facilitates the progression of glioma via regulation of KIF22/EGFR signaling

**DOI:** 10.1038/s41420-022-01018-0

**Published:** 2022-04-18

**Authors:** Yeqing Ren, Biao Yang, Geng Guo, Jianping Zhang, Yanqi Sun, Dong Liu, Shihao Guo, Yongqiang Wu, Xiaogang Wang, Shule Wang, Wenju Zhang, Xiaolong Guo, Xuepeng Li, Ren Li, Jianhang He, Zihan Zhou

**Affiliations:** 1 Department of Neurosurgery, The First Hospital, Shanxi Medical University, Taiyuan, Shanxi Province P. R. China; 2 Shanxi Provincial Key Laboratory of Brain Science and Neuropsychiatric Diseases, Taiyuan, Shanxi Province P. R. China; 3 Department of Neurosurgery, Liuzhou People’s Hospital, Guangxi Medical University, Liuzhou, Guangxi Province P. R. China

**Keywords:** Cancer genetics, Oncogenes

## Abstract

Identifying the mechanism of glioma progression is critical for diagnosis and treatment. Although studies have shown that guanylate-binding protein 2(GBP2) has critical roles in various cancers, its function in glioma is unclear. In this work, we demonstrate that GBP2 has high expression levels in glioma tissues. In glioma cells, depletion of GBP2 impairs proliferation and migration, whereas overexpression of GBP2 enhances proliferation and migration. Regarding the mechanism, we clarify that epidermal growth factor receptor (EGFR) signaling is regulated by GBP2, and also demonstrate that GBP2 interacts directly with kinesin family member 22(KIF22) and regulates glioma progression through KIF22/EGFR signaling in vitro and in vivo. Therefore, our study provides new insight into glioma progression and paves the way for advances in glioma treatment.

## Introduction

Glioma is among the most frequently occurring types of adult primary brain cancer, with a very high mortality rate owing to its localization and strong invasion capacity [1–3]. Despite treatment with surgical resection, chemotherapy, and radiation therapy, the median survival time of glioma patients is only 14–16 months [4], death from glioma accounts for the major death of primary brain cancers [1].

At least four subtypes of human gliomas have been described: pro-neural, neural, classical, and mesenchymal subtypes [1, 5]. Recently, substantial progress has been made regarding our understanding of the molecular pathogenesis of gliomas. Distinct proteins have been identified in different glioma subtypes, including YKL40 [5, 6], fibronectin 1 (FN1) [7], EGFR [5–7], and Stat3 [8]. These advances have been used both to improve diagnostics and to generate more effective therapeutic strategies, such as targeted therapies, for gliomas. However, it is essential to identify even more potential target pathways.

GBPs (guanylate-binding proteins), which were first found to be induced by IFN-γ in human fibroblasts, belong to the superfamily of interferon-inducible GTPases [9]. GBP2, a member of the GTPase superfamily, has critical roles in carcinoma. Studies have demonstrated that GBP2 suppresses cell proliferation in colorectal cancer via WNT signaling [10]. In breast cancer, GBP2 inhibits mitochondrial fission and cell invasion [11], and GBP2 overexpression is correlated with a better prognosis [12]. However, high expression of GBP2 is an indicator of esophageal squamous cell carcinoma (ESCC) [13]. It was also reported that GBP2 promotes invasion of GBM via the Stat3/FN1 cascade [14]. GBP2 has also been shown to be a prognostic biomarker of pancreatic adenocarcinoma [15]. Thus, GBP2 also has different functions in different tissues.

KIF22 belongs to the kinesin-10 family and plays diverse parts in cytoskeleton dynamics and during synaptic development [16–19]. Recently, studies have provided insight into the role of KIF22 during cancer progression. For example, KIF22 regulates EGFR signaling and attenuates the internalization of lung cancer cells [17]. In prostate cancer patients, high KIF22 expression is correlated with tumor development and poor prognosis [20]. Studies of KIF22 functions in breast cancer, gastric cancer, and tongue SCC have also been reported [21–23].

This work showed that GBP2 promoted proliferation and migration in glioma cells by regulating EGFR signaling pathway through interactions with KIF22. In vivo, depletion of GBP2 reduced tumor sizes. In summary, we clarified the oncogenic role of GBP2 in glioma and elucidated for the first time the GBP2/KIF22/EGFR pathway in glioma progression.

## Results

### GBP2 was highly expressed in glioma

**Fig. 1:**
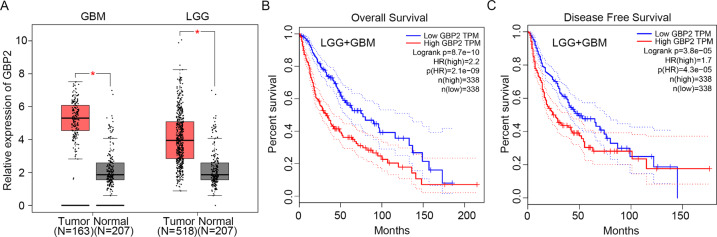
GBP2 is highly expressed in glioma. **A** GBP2 expression is highly elevated in glioma cells. Analysis of GBP2 expression in GBM and LGG from The Cancer Genome Atlas (TCGA) database. **B** Overall survival in patients with different GBP2 expression levels. **C** Disease-free survival in patients with different GBP2 expression levels.

The expression of GBP2 was detected in GBM and LGG samples; the results showed that mRNA levels of GBP2 in GBM and LGG were higher than those in normal controls (three times higher in GBM and double in LGG, Fig. 1A). With respect to overall survival and disease-free survival, lower GBP2 expression was associated with a higher survival percentage in LGG combined with GBM samples (Fig. 1B, C).

### Knockdown of GBP2 impaired proliferation and migration of glioma cells

**Fig. 2:**
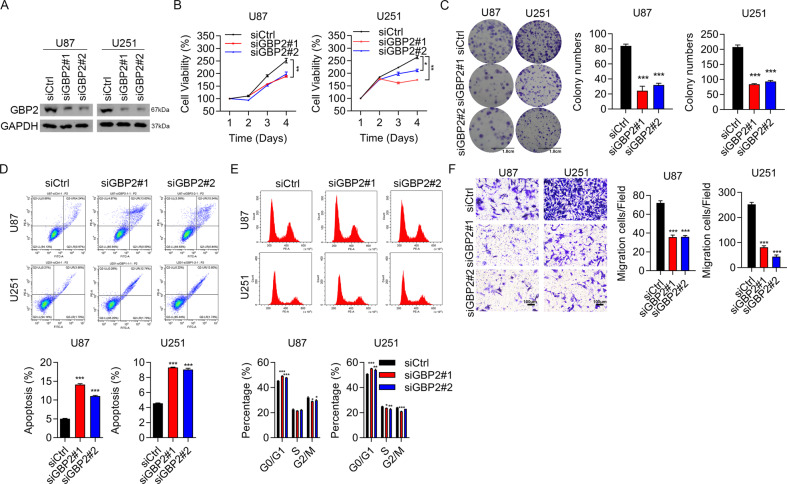
Depletion of GBP2 impairs proliferation and migration in glioma cells. **A** GBP2 was depleted efficiently. Western blotting results for GBP2 knockdown in U87 and U251 cells. **B** CCK8 assay results for cell proliferation in U87 and U251 cells transfected with siCtrl, siGBP2#1, or siGBP2#2. **C** Colony formation assay results of U87 and U251 cells transfected with siCtrl, siGBP2#1, or siGBP2#2. Clusters of more than 50 cells were identified as colonies. **D** Apoptosis results for U87 and U251 cells transfected with siCtrl, siGBP2#1, or siGBP2#2 using annexin V and PI. **E** Cell cycle results for U87 and U251 cells transfected with siCtrl, siGBP2#1, or siGBP2#2 using PI staining. **F** Transwell assay results for U87 and U251 cells transfected with siCtrl, siGBP2#1, or siGBP2#2. Each experiment repeated as least 3 times. Magnification: ×10. *P < 0.05, ^**^P < 0.01, ^***^P < 0.001.

Western blotting confirmed the high depletion efficiency of GBP2 in both glioma cell types (Fig. 2A). Cell proliferation was analyzed by CCK8 assay. With GBP2 depletion, cell viability was significantly reduced in both cells over 4 days (Fig. 2B). In the siGBP2 group, colony numbers decreased by at least half compared with those of the control group (Fig. 2C). We also carried out flow cytometry assays. The results showed that the percentage of apoptosis cells was nearly doubled by GBP2 depletion (Fig. 2D). Knockdown of GBP2 increased the proportion of G_0_/G_1_-phase cells in the cell cycle (Fig. 2E). Depletion of GBP2 apparently impaired cell migration by at least 50% (Fig. 2F). All the above results confirmed the oncogenic role of GBP2.

### Overexpression of GBP2 enhanced proliferation and migration of glioma cells

**Fig. 3:**
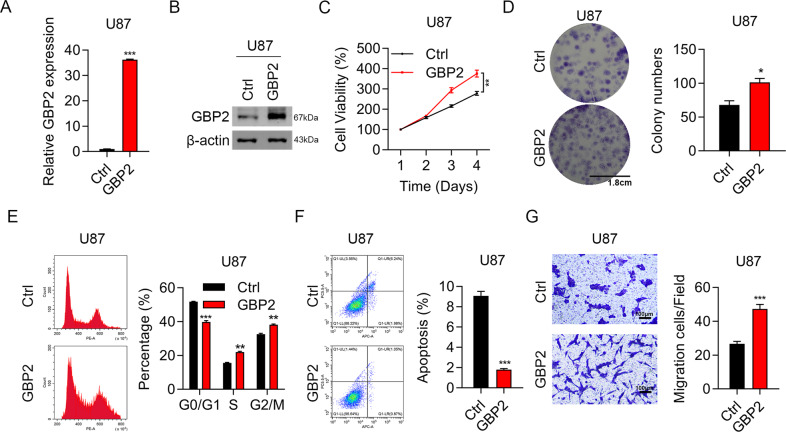
Overexpression of GBP2 enhances proliferation and migration in glioma cells. **A** RT-qPCR results confirming overexpression of GBP2 at the mRNA level. **B** Western blotting results confirming overexpression of GBP2 at the protein level. **C** CCK8 analysis of cell proliferation in GFP- and GFP-GBP2-overexpressing U87 cells. **D** Colony formation analysis in GFP- and GFP-GBP2-overexpressing U87 cells. **E** Cell cycle analysis of U87 cells overexpressing GFP or GFP-GBP2 using PI staining. **F** Apoptosis analysis of GFP- and GFP-GBP2-overexpressing U87 cells using annexin V and PI. **G** Transwell invasion results of U87 cells overexpressing GFP or GFP-GBP2. Each experiment repeated as least 3 times. Magnification: ×10. ^*^P < 0.05. ^**^P < 0.01, ^***^P < 0.001.

The RT-qPCR and western blotting results showed that GBP2 was highly expressed in U87 cells (Fig. 3A, B). The cell viability assay showed that overexpression of GBP2 enhanced cell viability (Fig. 3C). Colony formation improved by a factor of at least two in GBP2-overexpressing cells (Fig. 3D). Flow cytometry analysis demonstrated that GBP2 overexpression accelerated the cell cycle (the proportions of S- and G_2_/M-phase cells increased, while those of G_0_/G_1_-phase cells decreased) (Fig. 3E) and attenuated cell apoptosis (by nearly 80%) (Fig. 3F). The numbers of migration cells nearly doubled in GBP2-overexpressing U87 cells (Fig. 3G). Therefore, the results of the overexpression experiments were highly consistent with those of the knockdown experiments.

### GBP2 regulated the EGFR signaling pathway

**Fig. 4:**
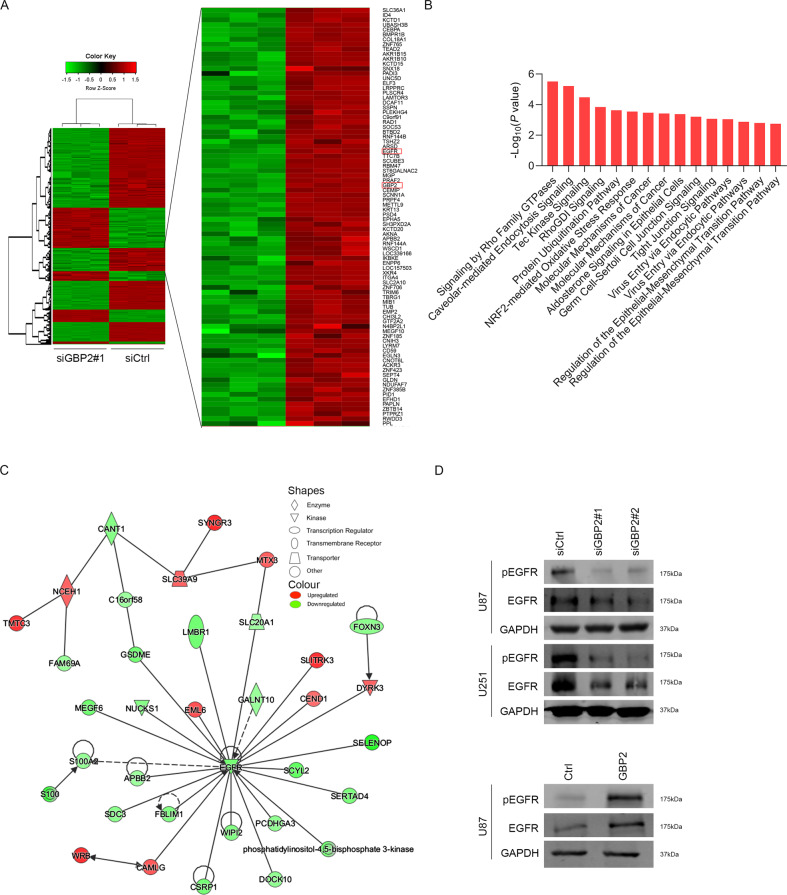
GBP2 regulates EGFR signaling pathway. **A** Heat map showing distinct level of mRNAs in control and siGBP2 cells. In the clustering analysis, red indicates upregulated genes, green indicates downregulated genes, and gray dots represent genes showing no change in expression. **B** The pathway enrichment analysis using IPA analysis with GBP2 knockdown according to microarray datasets. **C** IPA analysis of biological protein interactions with GBP2 involved in EGFR signaling pathway according to microarray datasets. **D** Western blotting results showing changes in protein levels in the EGFR signaling pathway. U87 cells with GBP2 overexpression or depletion are analyzed.

To further clarify the mechanism, gene microarray was performed to search for differentially expressed genes after GBP2 depletion. The results showed that 243 mRNAs were upregulated, while 560 mRNAs were downregulated (Fig. 4A). To identify the role of GBP2 in glioma progression, IPA analysis was performed based on the microarray data. Canonical_Pathway analysis showed that the at least 10 pathways were significantly enriched, including Signaling by Rho Family GTPases pathway and RhoGDI Signaling et al (Fig. 4B). While the network analysis indicated that EGFR as the target of GBP2. EGFR and its related genes were upregulated after GBP2 knockdown, which included APBB2, C16orf58, CAMLG, CANT1, CEND1, CSRP1, DOCK10, DYRK3, EML6, FAM69A, FBLIM1, FOXN3, GALNT10, GSDME, LMBR1, MEGF6, MTX3, NCEH1, NUCKS1, and PCDHGA3 (Fig. 4C). To further validate the results, we checked the levels of key proteins in the EGFR pathway from two Glioma cell lines through western blotting. Although both the p-EGFR and EGFR were downregulated upon GBP2 depletion, the ratio of pEGFR/EGFR was also reduced. Consistent results were observed in GBP2 overexpression experiments (Fig. 4D and Supplementary Fig. 1). Therefore, GBP2 regulates the EGFR signaling pathway.

### GBP2 interacted with KIF22 and regulated KIF22 expression

**Fig. 5:**
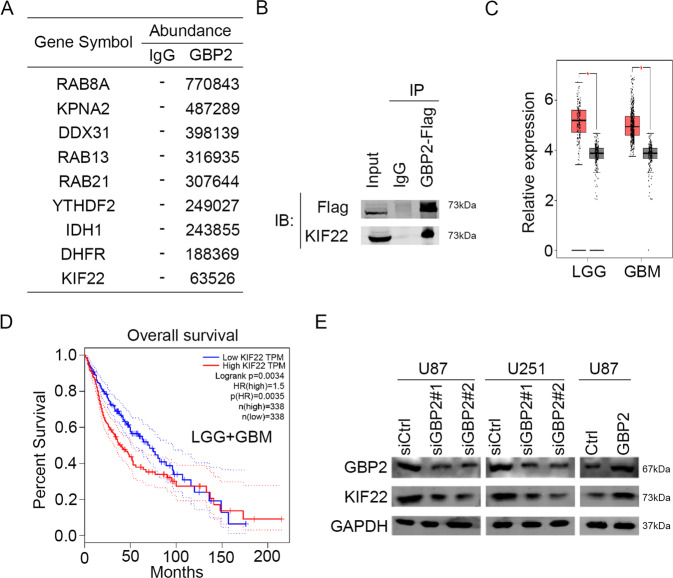
GBP2 interacts with KIF22 and regulates KIF22 expression. **A** Proteins with large differences in expression levels between control and GBP2-overexpressing cells. **B** Co-immunoprecipitation results showing the interaction between GBP2 and KIF22. **C** Analysis of KIF22 expression in GBM and LGG from TCGA database. **D** Overall survival in patients with different KIF22 expression levels. **E** Western blotting results showing that GBP2 regulates the expression level of KIF22. Each experiment repeated as least 3 times.

In order to explore the molecular mechanism of GBP2 regulating EGFR activity, we employed mass spectrometry to search for proteins directly interacting with GBP2. We found that 9 proteins were particularly interacted with GBP2 (Fig. 5A). Among the 9 proteins, we found that KIF22, DDX31 and YTHDF2 could regulated the expression and activity of EGFR [17, 24, 25]. In turn, we selected them for further investigation. As the co-IP results indicated, GBP2 could interact with KIF22 in glioma cells, but not DDX31 and YTHDF2 (Fig. 5B and Supplementary Fig. 2). Previously study showed that KIF22 delayed EGFR internalization and enhanced EGFR signaling. We then analyzed the expression of KIF22 in GBM and LGG, and found identical results to those obtained for GBP2 (Fig. 5C). In the overall survival analysis, lower KIF22 expression was associated with a higher survival percentage (Fig. 5D). In both cell types, knockdown of GBP2 significantly downregulated the expression of KIF22. Consistently, GBP2 overexpression upregulated KIF22 protein levels (Fig. 5E).

### Knockdown of KIF22 suppressed proliferation and migration of glioma cells

**Fig. 6:**
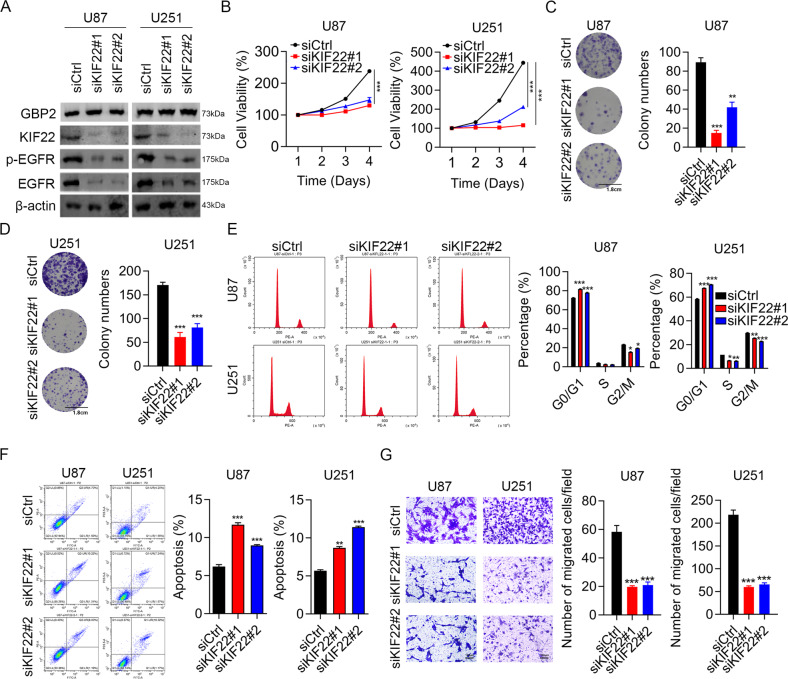
Knockdown of KIF22 impaired proliferation and migration in glioma cells. **A** Western blotting results confirming KIF22 knockdown efficiency, and showing expression of proteins related to EGFR signaling pathway in U87 and U251 cells transfected with siCtrl, siKIF22#1, or siKIF22#2. **B** CCK8 assay results for U87 and U251 cells transfected with siCtrl, siKIF22#1, or siKIF22#2. **C**, **D** Colony formation assay results for U87 and U251 cells transfected with siCtrl, siKIF22#1, and siKIF22#2. **E** Cell cycle assay results for U87 and U251 cells transfected with siCtrl, siKIF22#1, or siKIF22#2 using PI staining. **F** Apoptosis assay results for U87 and U251 cells using annexin V and PI staining. **G** Transwell invasion assay results for U87 and U251 cells. Each experiment repeated as least 3 times. Magnification: ×10. *P < 0.05, **P < 0.01, ***P < 0.001.

Several studies have identified KIF22 as an oncogene [17, 20–23]. Here, we explored the function of KIF22 in glioma cells. The knockdown efficiency of siKIF22 was confirmed by western blotting. KIF22 is required for the EGFR signaling pathway. Levels of proteins related to the EGFR signaling pathway were significantly decreased after KIF22 depletion in the U87 and U251 cell lines, while GBP2 expression not altered after KIF22 knockdown (Fig. 6A), and cell viability was reduced by at least half in both cell types (Fig. 6B). Colony formation numbers decreased significantly in KIF22-knockdown cells (Fig. 6C, D). The cell cycle analysis showed that knockdown of KIF22 blocked glioma cells at G0/G1 phase (Fig. 6E). The percentage of apoptotic cells nearly doubled following KIF22 depletion (Fig. 6F). Invasion ability was also suppressed, decreasing by at least 60% compared with that of control cells (Fig. 6G). All the above results were consistent with those obtained for GBP2 and indicated a role for KIF22 as an oncogene in glioma cells.

### GBP2 regulated EGFR signaling and glioma progression dependent on KIF22

**Fig. 7:**
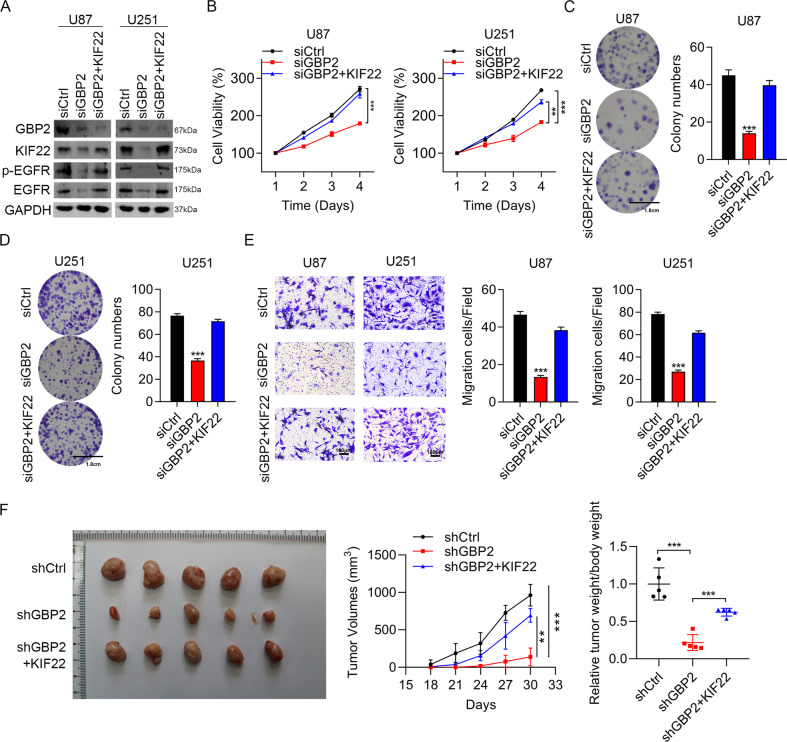
GBP2 regulates glioma progression through KIF22. **A** Western blotting results confirming GBP2 knockdown efficiency and showing expression of EGFR, p-EGFR and KIF22 in U87 and U251 cells transfected with siCtrl, siGBP2, or siGBP2 + KIF22. **B** Cell viability assay results for U87 and U251 cells transfected with siCtrl, siGBP2, or siGBP2 + KIF22. **C**, **D** Colony formation assay results for U87 and U251 cells transfected with siCtrl, siGBP2, or siGBP2 + KIF22. Clusters of more than 50 cells were identified as colonies. **E** Transwell assay results for U87 and U251 cells transfected with siCtrl, siGBP2, or siGBP2 + KIF22. **F** U87 cells exogenously expressed shCtrl, shGBP2, and shGBP2 + KIF22 were injected into athymic nude mice (*n* = 5), and tumor formation was analyzed. Representative images and weight of xenografted tumors are shown. Each experiment repeated as least 3 times. Magnification: ×10. **P < 0.01, ***P < 0.001.

The western blotting results demonstrated that the downregulation of EGFR and p-EGFR caused by GBP2 depletion was rescued by KIF22 overexpression (Fig. 7A). Cell viability and colony formation numbers suppressed by GBP2 knockdown returned nearly to normal levels when overexpression of KIF22 was introduced (Fig. 7B–D). Impaired migration ability was also rescued by KIF22 overexpression in both glioma cell lines (Fig. 7E). We also measured the in vivo tumorigenicity. Consistent with the above results, whereas tumor size was reduced by GBP2 knockdown, it was restored to control levels by KIF22 overexpression (Fig. 7F). Thus, GBP2 regulated glioma progression dependent on KIF22.

## Discussion

To the best of our knowledge, this study was the first time to elucidate the GBP2/KIF22/EGFR pathway in the progression of glioma carcinoma. We employed knockdown and overexpression strategies, and the results of in vitro and in vivo assays were highly consistent in demonstrating the function of GBP2 and KIF22 as oncogenes in glioma.

Despite great progress over the past decades in determining the molecular pathogenesis of gliomas [5–8], the mortality rate of glioma patients has not been significantly reduced. To improve the prognosis of glioma patients, early diagnosis, reliable prognostic biomarkers, and targeted therapy strategies are needed. Potential targets including YKL40, FN1, EGFR, and Stat3 have been discovered [5–8]. However, more targets are needed. In this work, we identified GBP2/KIF22/EGFR as a potential therapeutic target for glioma. Since several other KIF superfamily members have been reported to be involved in the development of glioma with low and high grades [16, 26], and KIF proteins targeting drugs were in clinical trials in other pathologies and could give novel therapeutic possibilities in the future. Nevertheless, the detailed mechanisms, as well as the potential biomarker need to be further investigated. Thus, our study will pave the way for further advances in glioma diagnosis and treatment.

GBP2, a member of the GTPase superfamily, has functions in protective immunity [27]. Recently, studies have focused on the functions of GBPs in cancer progression. GBP2 is highly expressed in esophageal SCC, where it represents a potential biomarker [13]. Yu et al. demonstrated that GBP2 was highly expressed in human glioma and promoted the invasion of glioma cells [14], which was consistent with our study. Besides, we also indicated that GBP2 played a critical role in glioma proliferation. Otherwise, Yu et al. also suggested that Stat3/FN1 signaling pathway was a key point in the glioma invasion regulated by GBP2. Silencing FN1 or blocking Stat3 activity by WP1066 could reversed the invasiveness induced by GBP2. In our study, microarray assay and subsequent experiment indicated that GBP2 knockdown could significantly depress the expression and the phosphorylation of EGFR. In glioblastoma, classical subtype is characterized with EGFR amplification. EGFR plays critical roles in many cellular processes, such as proliferation, migration, adhesion [28], as well as tumor growth [29]. Further work will include determining relationship between these two pathways, and whether they exist in the same GBM stage or at different stages.

To explain the role of EGFR activity regulated by GBP2, the mass spectrometry was performed. The result showed that KIF22 was more abundant in the GBP2-Flag group compared than IgG group. The following CO-IP assay and Western blot suggested that the GBP2 interacted with KIF22 and regulated the expression of KIF22. In addition, our results indicated that the protein level of KIF22 was decreased after GBP2 knockdown, while no significant changes were found in the mRNA level, as indicated by RNA-seq, which might be owing to that GBP2 regulated KIF22 expression at post-transcriptional level. KIF22 has been reported essentially for kinds of cancer progression. Importantly, Pike R et al. reported that high expression of KIF22 maintained the location of EGFR at cytomembrane, leading the continuous activation of EGFR [17]. Similarly, our study indicated that silencing the KIF22 significantly suppressed the progression of glioma progression, and also impaired the protein expression and phosphorylation of EGFR. These results suggested that KIF22 is essentially for EGFR activation in glioma cells.

Furthermore, to confirm whether GBP2 promoted the glioma progression by KIF22, we overexpressed KIF22 in GBP2 knockdown cells. As expected, KIF22 overexpression markedly reversed the inhibited malignant phenotype induced by GBP2 silencing in vitro and in vivo. These results confirmed that GBP2 regulated glioma progression by KIF22/EGFR signaling pathway. However, how does GBP2 regulate KIF22 protein level under the condition of binding to KIF22 needs further studies.

In summary, we demonstrate that GBP2 was overexpressed in glioma tissues. Depletion of GBP2 impaired the proliferation and migration, whereas overexpression of GBP2 showed the opposite effect. Regarding the mechanism, we clarify that EGFR signaling was regulated by GBP2, and also demonstrated that GBP2 directly interacted with KIF22 and regulated glioma progression through KIF22/EGFR signaling. Therefore, our study provides new insight into glioma progression and paves the way for advances in glioma treatment.

## Materials and methods

### Clinical samples form TCGA

The expression of GBP2 and KIF22 were analyzed in GBM or LGG (TCGA data) and normal tissues (Match TCGA normal and GTEx data) in the website (http://gepia.cancer-pku.cn). Moreover, the overall survival and disease-free survival was also analyzed in the website (http://gepia.cancer-pku.cn).

### Cell culture and cell transfection

Glioma cell lines U87 and U251 were cultured in Dulbecco’s modified Eagle medium (DMEM) (Gibco), with 10% fetal bovine serum (FBS) (Gibco) and 2 mM glutamine, and maintained in a 37 °C incubator containing 5% CO_2_. Plasmids were constructed for overexpression of GBP2 (pEGFP-C1-GBP2 and pcDNA3.1-NFlag-GBP2) and KIF22 (pcDNA3.1-NFlag-KIF22). Small interfering RNAs (siRNAs) siCtrl: 5′-UUCUCCGAACGUGUCACGU-3′, siGBP2#1: 5′-GCCAGAACACACCCUAGUU-3′, siGBP2#2: 5′-CCAAAUGUUCCAGAGGAAA-3′, siKIF22#1: 5′-GGUCCAAGGAGGUGAUCAATT-3′, and siKIF22#2: 5′-AAGCAAGAUUGGAGCUACUCGUCTT-3′ were ordered from Hippo Biotechnology (Huzhou, China). Lentiviral short hairpin RNA plasmids pLKO.1-shGFP (CAAGCTGACCCTGAAGTTCAT) and pLKO.1-GBP2 (ATTGAAGTGGAACGTATAAAG) were purchased from Sigma-Aldrich. Lipofectamine RNAiMAX transfection reagent (Invitrogen) was used for transfection of siRNA following the manufacturer’s protocol. For overexpression plasmids, Lipofectamine 3000 (Invitrogen) was used.

### RNA extraction, reverse transcription, and quantitative real-time PCR (RT-qPCR)

Total RNA in U87 and U251 cells was extracted with TRIzol reagent (Invitrogen). The RNA was reverse transcribed using the reverse transcription kit (Promega), and RT-qPCR assays were performed with SYBR Master Mix (TaKaRa) on an Agilent MX3000p real-time PCR system. The following primers were used: GBP2 forward, 5′-GATTGGCCCGCTCCTAAGAA-3′ and reverse, 5′-TTGACGTAGGTCAGCACCAG-3′; β-actin forward, 5′-CATGTACGTTGCTATCCAGGC-3′ and reverse, 5′-CTCCTTAATGTCACGCACGAT-3′.

### Cell viability assay

Cell viability was evaluated using a Cell Counting Kit-8 (CCK-8) purchased from Sigma. Cells were digested with trypsin and seeded in triplicate into 96-well plates at a density of 2000 cells with 100 µL culture medium per well. After incubation for the appropriate number of days, 10 µL CCK8 reagent was added to cells with continuous incubation at 37 °C for 2 h. The absorbance of each well was measured at 450 nm. Each treatment groups included at least five wells.

### Colony formation

U87 or U251 cells (1 × 10^3^ cells per well) were seeded into 6-well plates. The medium was changed every 3 days. Colonies were formed after 14 days. Cells were washed with phosphate-buffered saline (PBS) three times, fixed with 4% paraformaldehyde for 20 min, and stained with GIEMSA staining solution for 20 min. More than 50 cells were considered to constitute a colony. The colonies were counted under a optical microscope (AxioObserverZ1) to capture digital images from four random microscope fields. Then, the colonies were counted using ImageJ software (version1.47, USA), each treatment groups included at least triplicate wells.

### Cell cycle analysis

Following the manufacturer’s manual, the cell cycle was analyzed using FxCycle PI/RNase Staining Solution (Invitrogen). First, cells grown to about 80% confluence were digested with trypsin and centrifuged for 5 min at 13000 rpm. Iced D-Hanks (pH = 7.2–7.4) buffer was used to wash the pellets, and iced 75% ethanol was used to fix the cells for at least 2 h. After centrifugation and washing with D-Hanks, 0.5 mL of FxCycle™ PI/RNase staining solution was added, and the cells were incubated at room temperature for 15–30 min. Finally, samples were analyzed with 488 nm excitation, and emission was collected with a 585/42 bandpass filter or equivalent. Images are representative of three different experiments.

### Cell apoptosis assay

Cell apoptosis of U87 and U251 cells was evaluated by Annexin V-FITC and propidium iodide (PI). Cell suspensions were collected and centrifuged at 13,000 rpm for 5 min, then washed with 200 µL 1× binding buffer and resuspended in 190 µL 1×binding buffer. PI (10 µL, 20 µg/mL) was added to the samples and they were analyzed by flow cytometry. Each treatment groups included at least triplicate wells.

### Cell migration assay

Cells (2.5 × 10^4^ per insert) were seeded in the upper chamber of a Corning transwell chamber (8 µm pore size). The lower chamber was filled with DMEM with the addition of 10% FBS as a chemical attractant. The migration of cells was measured. Crystal violet (0.1 μM) was used to stain cells for 30 min. Images of migrant cells from 6 random fields were collected under an optical microscope (AxioObserverZ1). Each treatment groups included at least triplicate wells, the results are represented in migrated cells/field.

### Western blotting

Total protein was lysed with RIPA buffer containing protease and phosphatase inhibitors cocktail. A BCA Protein Assay Kit was used to measure protein concentration. Equal amounts of proteins were subjected to sodium dodecyl sulfate polyacrylamide gel electrophoresis (SDS-PAGE), followed by transfer to polyvinylidene fluoride membranes (Millipore), and blocked with 5% non-fat milk dissolved in Phosphate Buffered Saline with Twen-20 (PBST) for 1 h at room temperature. Primary antibodies were added to the membranes and incubated overnight at 4 °C. After incubation with peroxidase-conjugated secondary antibodies, protein abundance was analyzed using an enhanced chemiluminescence system (Millipore). The primary antibodies were used as followed: anti-GBP2 (Abcam, ab179829, 1:1000), anti-GAPDH (Abcam, ab9485, 1:1000), anti-β-actin (Abcam, ab8227, 1:1000), anti-phospho-EGFR (CST, #3777, 1:1000), anti-EGFR (Abcam, ab52894, 1:1000), and anti-KIF22 (Abcam, ab75783, 1:1000), anti-YTHDF2 (Proteintech, 24744-1-AP) and anti-DDX31(Abclonal: A15892).

### Xenograft model

PBS (0.1 ml) containing 1 × 10^7^ U87 cells transfected with shRNA, shGBP2 or shGBP2 + KIF22 overexpression were injected subcutaneously into the left posterior flank area of each mouse, and the tumor size was evaluated twice a week using a caliper. Athymic nude mice, 5–6 weeks old, were randomly divided into three groups, with five mice per group. The mice were sacrificed after 30 days. Vernier calipers were used to measure the length and width of the tumor, and the tumor volume was calculated according to the following formula: *V* = 1/2*ab^2^ (*a*, length; *b*, width). This study was approved by The First Hospital of Shanxi Medical University Animal Ethics Committee.

### Co-immunoprecipitation experiment and mass spectrometry

FLAG-tagged GBP2 or KIF22 was overexpressed in 293T cells and lysed with a low-salt lysis buffer supplemented with proteinase inhibitors and phosphatase inhibitors. Immunoprecipitations were carried out using 1 mg of whole-cell extract and 3 µL of α-FLAG antibody. Immunoprecipitations were incubated for 2 h at 4 °C. Samples were washed with 1 mL immunoprecipitation buffer three times, and detected by mass spectrometry analysis or SDS-PAGE. The protein derived from co-IP assay were detected using mass spectrometry assay. Briefly, equal amounts of proteins were loaded and separated in 12% SDS-PAGE gels. The gels were stained with Coomassie blue (SimplyBlue SafeStain, Invitrogen) overnight before excised into slices. Then, the protein bands of interest were excised and the gels were de-stained and digested with trypsin at 37 °C overnight. The digested proteins were then desalted for LC-MS/MS analysis (Genechem, Shanghai) according to previous studies indicated [30]. The proteins were identified and quantitated by Protein Pilot 4.0TM software (AB Sciex, USA).

### Microarray

TRIzol reagent (Invitrogen) was used to extract total RNA, and agarose electrophoresis and a NanoDrop instrument (Thermo) were used for quality control and quantification. Microarray analysis was performed by Shanghai GeneChem Co., Ltd. (Shanghai, China). The dysregulated gene was identified by *p* < 0.05 and fold change >2. Moreover, the gene expression network was performed by Ingenuity Pathway Analysis (IPA).

### Statistical analysis

Data were expressed as mean ± standard deviation, and analyzed using GraphPad Prism 8.0. Differences between two groups were analyzed by two-tailed Student’s *t* test. Differences among more than two groups were analyzed by one-way ANOVA followed by Tukey’s post hoc test. *P* < 0.05 was considered to indicate statistically significant differences.

## Supplementary information

Supplementary Figure

WB uncropped imagine

## Data Availability

All data generated or analyzed during this study are included in this published article.

## References

[1] Weller M, Wick W, Aldape K, Brada M, Berger M, Pfister S (2015). Glioma. Nat Rev Dis Prim.

[2] Ferlay J, Parkin DM, Steliarova-Foucher E (2010). Estimates of cancer incidence and mortality in Europe in 2008. Eur J Cancer.

[3] Ostrom QT, Gittleman H, Liao P, Rouse C, Chen Y, Dowling J (2014). CBTRUS statistical report: primary brain and central nervous system tumors diagnosed in the United States in 2007-2011. Neuro Oncol.

[4] Louis DN, Perry A, Reifenberger G, von Deimling A, Figarella-Branger D, Cavenee WK (2016). The 2016 World Health Organization classification of tumors of the central nervous system: a summary. Acta Neuropathol.

[5] Verhaak RG, Hoadley KA, Purdom E, Wang V, Qi Y, Wilkerson MD (2010). Integrated genomic analysis identifies clinically relevant subtypes of glioblastoma characterized by abnormalities in PDGFRA, IDH1, EGFR, and NF1. Cancer Cell.

[6] Behnan J, Finocchiaro G, Hanna G (2019). The landscape of the mesenchymal signature in brain tumours. Brain.

[7] Brennan CW, Verhaak RG, McKenna A, Campos B, Noushmehr H, Salama SR (2013). The somatic genomic landscape of glioblastoma. Cell.

[8] Carro MS, Lim WK, Alvarez MJ, Bollo RJ, Zhao X, Snyder EY (2010). The transcriptional network for mesenchymal transformation of brain tumours. Nature.

[9] Cheng YS, Colonno RJ, Yin FH (1983). Interferon induction of fibroblast proteins with guanylate binding activity. J Biol Chem.

[10] Wang J, Min H, Hu B, Xue X, Liu Y (2020). Guanylate-binding protein-2 inhibits colorectal cancer cell growth and increases the sensitivity to paclitaxel of paclitaxel-resistant colorectal cancer cells by interfering Wnt signaling. J Cell Biochem.

[11] Zhang J, Zhang Y, Wu W, Wang F, Liu X, Shui G (2017). Guanylate-binding protein 2 regulates Drp1-mediated mitochondrial fission to suppress breast cancer cell invasion. Cell Death Dis.

[12] Godoy P, Cadenas C, Hellwig B, Marchan R, Stewart J, Reif R (2014). Interferon-inducible guanylate binding protein (GBP2) is associated with better prognosis in breast cancer and indicates an efficient T cell response. Breast Cancer.

[13] Guimaraes DP, Oliveira IM, de Moraes E, Paiva GR, Souza DM, Barnas C (2009). Interferon-inducible guanylate binding protein (GBP)-2: a novel p53-regulated tumor marker in esophageal squamous cell carcinomas. Int J Cancer.

[14] Yu S, Yu X, Sun L, Zheng Y, Chen L, Xu H (2020). GBP2 enhances glioblastoma invasion through Stat3/fibronectin pathway. Oncogene.

[15] Liu B, Huang R, Fu T, He P, Du C, Zhou W (2021). GBP2 as a potential prognostic biomarker in pancreatic adenocarcinoma. Peer J.

[16] Yu Y, Feng YM (2010). The role of kinesin family proteins in tumorigenesis and progression: potential biomarkers and molecular targets for cancer therapy. Cancer.

[17] Pike R, Ortiz-Zapater E, Lumicisi B, Santis G, Parsons M (2018). KIF22 coordinates CAR and EGFR dynamics to promote cancer cell proliferation. Sci Signal.

[18] Park SM, Littleton JT, Park HR, Lee JH (2016). Drosophila homolog of human KIF22 at the autism-linked 16p11.2 loci influences synaptic connectivity at larval neuromuscular junctions. Exp Neurobiol.

[19] Miki H, Okada Y, Hirokawa N (2005). Analysis of the kinesin superfamily: insights into structure and function. Trends Cell Biol.

[20] Zhang Z, Xie H, Zhu S, Chen X, Yu J, Shen T (2018). High Expression of KIF22/Kinesin-Like DNA Binding Protein (Kid) as a Poor Prognostic Factor in Prostate Cancer Patients. Med Sci. Monit.

[21] Yu ZY, Jiang XY, Zhao RR, Qin JJ, Luo CJ, Ren YX (2020). Effect of KIF22 on promoting proliferation and migration of gastric cancer cells via MAPK-ERK pathways. Chin Med J (Engl).

[22] Yu Y, Wang XY, Sun L, Wang YL, Wan YF, Li XQ (2014). Inhibition of KIF22 suppresses cancer cell proliferation by delaying mitotic exit through upregulating CDC25C expression. Carcinogenesis.

[23] Liu Y, Li RH, Ren G, Jiang J (2020). Suppression of KIF22 inhibits cell proliferation and xenograft tumor growth in tongue squamous cell carcinoma. Biomed Res Int.

[24] Daizumoto K, Yoshimaru T, Matsushita Y, Fukawa T, Uehara H, Ono M (2018). A DDX31/mutant-p53/EGFR axis promotes multistep progression of muscle-invasive bladder cancer. Cancer Res.

[25] Zhong L, Liao D, Zhang M, Zeng C, Li X, Zhang R (2019). YTHDF2 suppresses cell proliferation and growth via destabilizing the EGFR mRNA in hepatocellular carcinoma. Cancer Lett.

[26] Cho SY, Kim S, Kim G, Singh P, Kim DW (2019). Integrative analysis of KIF4A, 9, 18A, and 23 and their clinical significance in low-grade glioma and glioblastoma. Sci Rep.

[27] Vestal DJ, Jeyaratnam JA (2011). The guanylate-binding proteins: emerging insights into the biochemical properties and functions of this family of large interferon-induced guanosine triphosphatase. J Interferon Cytokine Res.

[28] Lemmon MA, Schlessinger J (2010). Cell signaling by receptor tyrosine kinases. Cell.

[29] Ladanyi M, Pao W (2008). Lung adenocarcinoma: guiding EGFR-targeted therapy and beyond. Mod. Pathol.

[30] Kang J, Zhao G, Lin T, Tang S, Xu G, Hu S (2013). A peptide derived from phage display library exhibits anti-tumor activity by targeting GRP78 in gastric cancer multidrug resistance cells. Cancer Lett.

