# Supplementary material for: GBP2 facilitates the progression of glioma via regulation of KIF22/EGFR signaling
Source: Cell Death Discov. 2022 Apr 18;8:208. doi: 10.1038/s41420-022-01018-0 (PMC9016070; doi:10.1038/s41420-022-01018-0)
Supplement: Supplementary file 1 — Supplementary Figure [file 41420_2022_1018_MOESM1_ESM.docx]

**Supplementary Figure 1. GBP2 regulates EGFR activity.**

**
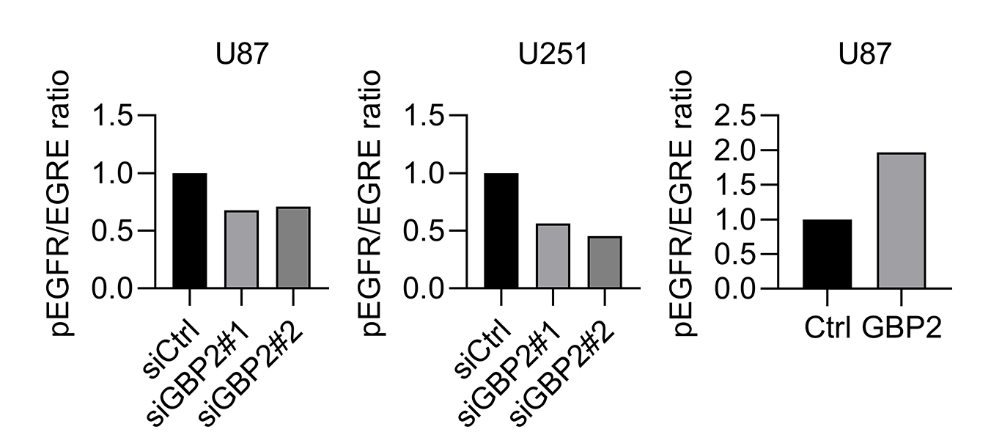
**

The ration of pEGFR/EGFR was quantified in glioma cells with GBP2 depletion or overexpression.

**Supplementary Figure 2.** **Co-immunoprecipitation assay to detect the interaction between GBP2 and DDX31 or YTHDF2.**

**
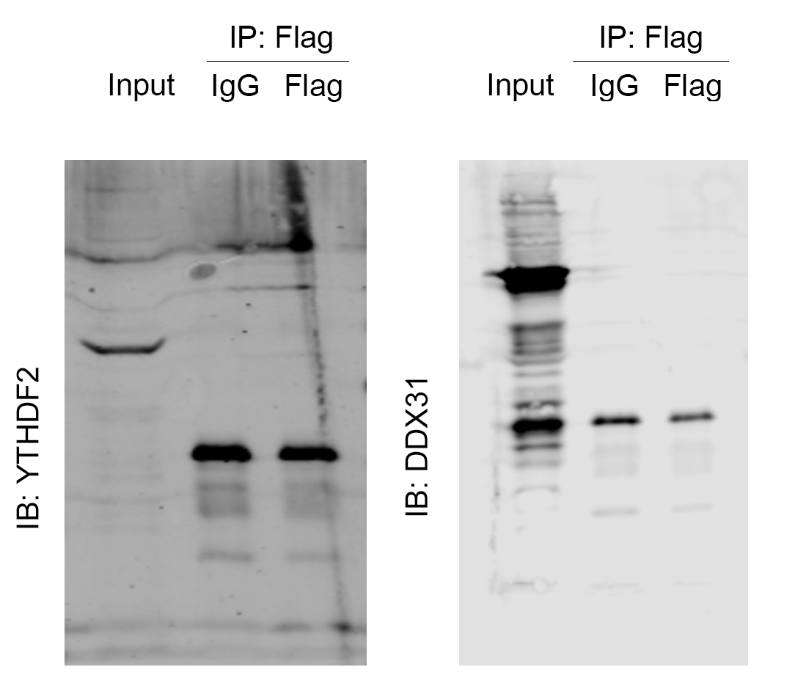
**

Co-immunoprecipitation results showed that the GBP2 could not interact with YTHDF2 and DDX31.
