# Supplementary figures and images for: GBP2 facilitates the progression of glioma via regulation of KIF22/EGFR signaling
Source: Cell Death Discov. 2022 Apr 18;8:208. doi: 10.1038/s41420-022-01018-0 (PMC9016070; doi:10.1038/s41420-022-01018-0)

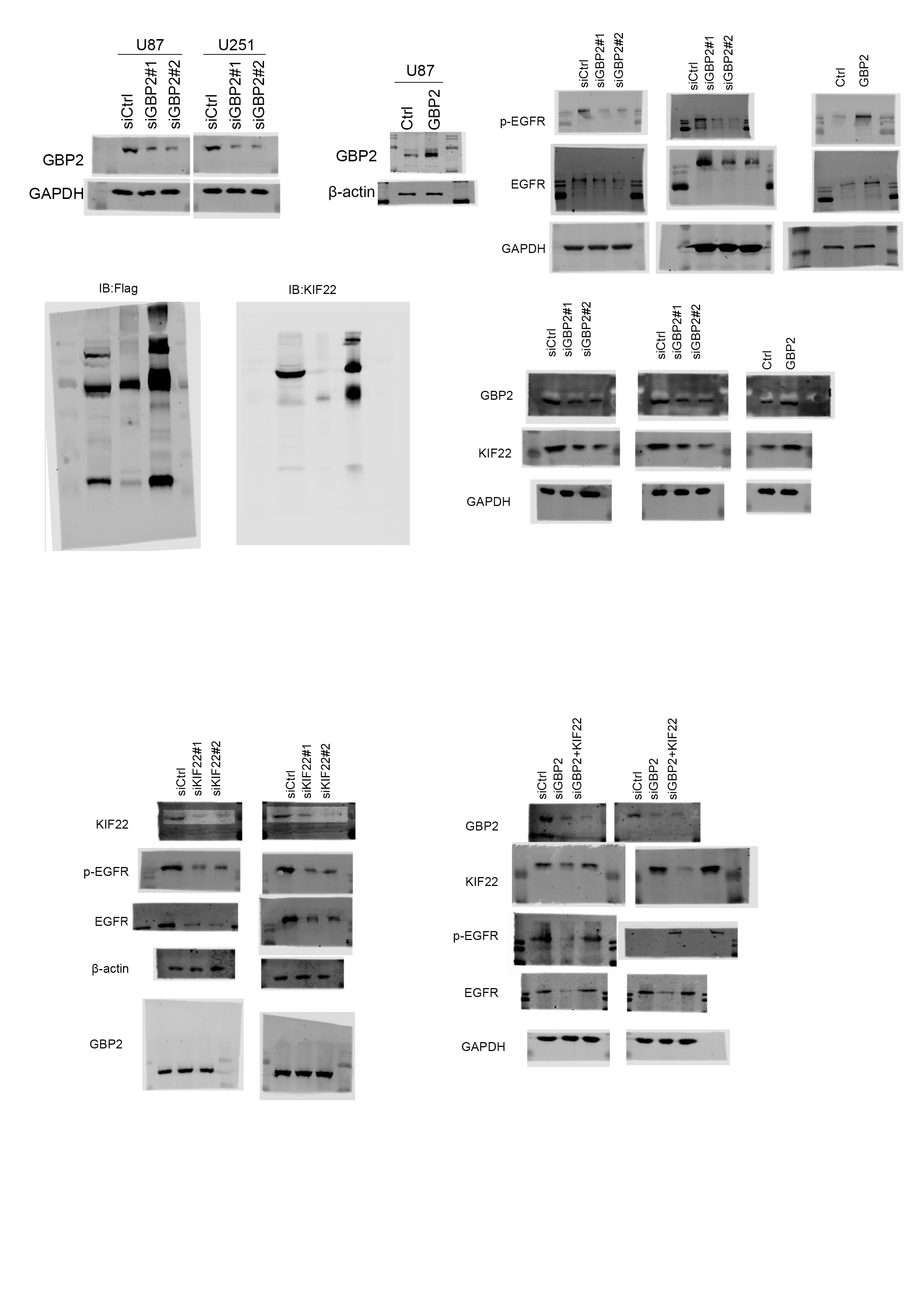

Supplement: Supplementary file 2 — WB uncropped imagine [file 41420_2022_1018_MOESM2_ESM.tif]
